# Medication adherence and health‐related quality of life among people with diabetes in Bangladesh: A cross‐sectional study

**DOI:** 10.1002/edm2.444

**Published:** 2023-07-25

**Authors:** Sabrina Ahmed, K. M. Saif‐Ur‐Rahman, Raja Ram Dhungana, Gantsetseg Ganbaatar, Fatema Ashraf, Yuichiro Yano, Katsuyuki Miura, M. S. A. Mansur Ahmed

**Affiliations:** ^1^ Department of Noncommunicable Diseases Bangladesh University of Health Sciences Dhaka Bangladesh; ^2^ NCD Epidemiology Research Center Shiga University of Medical Science Seta Tsukinowa‐Cho, Otsu Shiga Japan; ^3^ Center for Noncommunicable Diseases and Nutrition (CNCDN) James P Grant School of Public Health, BRAC University Dhaka Bangladesh; ^4^ Health Systems and Population Studies Division, icddr,b Dhaka Bangladesh; ^5^ College of Medicine, Nursing and Health Sciences University of Galway Galway Ireland; ^6^ Evidence Synthesis Ireland and Cochrane Ireland University of Galway Galway Ireland; ^7^ Faculty of Medicine, Nursing and Health Sciences Monash University Clayton Victoria Australia; ^8^ Department of Gynaecology and Obstetrics Shaheed Suhrawardy Medical College Dhaka Bangladesh

**Keywords:** determinants, diabetes, medication adherence, quality of life

## Abstract

**Introduction:**

Good adherence to anti‐diabetic medications is an important protective factor for decreasing diabetes‐related complications and disabilities but its association with health‐related quality of life (HRQoL) is understudied. The current study aimed to assess an association between medication adherence to anti‐diabetic drugs and HRQoL among people with diabetes in Dhaka city, Bangladesh.

**Methods:**

We conducted a cross‐sectional study among 480 people with diabetes aged between 50 and 70 years, who attended a tertiary‐level hospital in Dhaka city. We used the EuroQol‐5 Dimensions Questionnaire (EQ‐5D‐5L) to measure HRQoL and Morisky Medication Adherence Scale to assess the level of medication adherence to anti‐diabetic drugs. Multivariable logistic regression was performed to assess the significance of relevant factors.

**Results:**

The mean age of the participants was 59.0 (standard deviation [SD], 7.0) years. The majority of the participants (74%) had a lower level of medication adherence. The mean value of (EQ‐5D‐5L) was 2.0 (SD, 1.0). The percentage of severe disability in different domains were 6.7% for mobility, 3.5% for self‐care, 11.9% for usual daily activities, 11.9% for pain/discomfort and 11.3% for anxiety. After adjusting for age, sex, years of education, household expenditure, hypertension, duration of diabetes, glycemic status and multi‐morbidities; low adherence to anti‐diabetic medication was inversely associated with pain (OR, 0.26; 95% CI, 0.08–0.80; *p* = .036), and positively associated with anxiety (OR, 7.18; 95% CI, 1.03–9.59; *p* = .043).

**Conclusions:**

Low medication adherence to anti‐diabetic drugs was associated with anxiety and pain among the EQ‐5D‐5L indexes measured in people with diabetes in Dhaka, Bangladesh.

## Graphical abstract

In people with diabetes in Dhaka, Bangladesh, low medication adherence to anti‐diabetic drugs shows an association with anxiety and pain among the five dimensions of the quality of life index (EQ‐5D‐5L). Low adherence to anti‐diabetic medication was inversely associated with pain (OR, 0.26; 95% CI, 0.08–0.80; *p* = .036), and positively associated with anxiety (OR, 7.18; 95% CI, 1.03–9.59; *p* = .043).

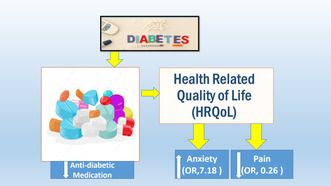

## 1 INTRODUCTION

Diabetes mellitus leading to global morbidity and mortality eventually causes an impaired quality of life among people with diabetes.^1^ South‐East Asia has 88 million people (aged 20–79 years) living with diabetes.^2^ In Bangladesh, diabetes is increasing at an alarming rate.^3^ Prevalence of diabetes was 8.1% among the Bangladeshi population in 2019 and is predicted to be 13% by 2030, and most of the people with diabetes have diabetes‐related complications.^2^, ^4^

The burden of diabetes on middle‐aged and older adults is increasing and related complications result in diminished health‐related quality of life (HRQoL).^5^ Recent studies including meta‐analysis revealed several factors related to low HRQoL among people with diabetes, such as low physical exercise, uncontrolled diabetes, the presence of multi‐morbidities, longer duration of diabetes, and depression.^6^, ^7^, ^8^ There were no different findings in studies conducted among Bangladeshi people with diabetes with similar interests.^5^, ^8^ Since life expectancy has risen drastically, catalysed by rapid infrastructural and economic expansion in Bangladesh, the elderly population is increasing. Age‐related physical disabilities are becoming a challenge for the country, resulting in decreasing quality of life.^9^ Regarding activities of daily living 10% of the people with diabetes have some sort of impairment reported by a recent study.^10^ However good adherence to anti‐diabetic medications is an important protective factor for decreasing diabetes‐related complications and disabilities.^11^, ^12^

Although low therapeutic adherence is common among people suffering from chronic diseases, especially among individuals with diabetes^13^; none of the earlier studies among the Bangladeshi population with diabetes have explored the association between adherence to anti‐diabetic medication and HRQoL. Therefore, this study aimed to investigate the association between medication adherence and HRQoL among people with diabetes in Bangladesh. Such knowledge is crucial for both preventive and clinical views for lowering the quality of life among individuals with diabetes.

## 2 PARTICIPANTS AND METHODS

### 2.1 Study participants and setting

For this particular research, we have conducted a hospital‐based cross‐sectional study in Dhaka, Bangladesh. The detail of the study methodology has been discussed elsewhere.^10^ In brief, the study period was between July to November 2017. We conducted the study at the Bangladesh Institute of Health Science (BIHS) hospital, which is a tertiary‐level hospital in Dhaka that especially deals with diabetes patients through diabetes care services. The study population was patients diagnosed with diabetes mellitus attending the selected facility for inpatient and outpatient services, both sexes and aged between 50 and 70 years (*n* = 480). For deciding the sample size, we used the following formula, *n* = *z* ^2^ *p* (1 − *p*)/*d* ^2^; where *n* = desired sample size. *z* = Standard error of the mean which corresponds to 95% confidence level (1.96), *p* = probability of condition being studied (0.50), and *d* is precision (corresponding to effect size). After considering the non‐response rate of 20%, the final sample size was calculated as 461. We initially selected 500 participants purposively, however after excluding psychologically compromised (cannot follow instructions), *n* = 5, and severely ill individuals (*n* = 15), the remaining 480 participants were analysed for the present study.

Written informed consent was obtained from all participants prior to the study. The study followed the code of ethics of the World Medical Association (1975 Declaration of Helsinki). The study obtained approval from the Institutional Review Board of the Bangladesh University of Health Science.

### 2.2 Measurement of Quality of Life (EQ‐5D‐5L Score)

The HRQoL was measured by the EuroQol‐5 Dimensions Questionnaire (EQ‐5D‐5L)^14^ and medication adherence to anti‐diabetic drugs was measured using Morisky Medication Adherence Scale.^15^, ^16^ The EQ‐5D‐5L is a commonly used measure of the quality of life used for instrumental calculating of quality‐adjusted life‐years (QALYs). It contains five domains (mobility, self‐care, usual activities, pain or discomfort and anxiety or depression), with each having five levels (no problems, some problems, moderate problems, severe problems or extreme problems).^17^, ^18^ This instrument can be self‐administered, and respondents also rate their overall health on the day of the interview on a 0–100 hash‐marked, vertical visual analogue scale known as the EuroQol‐visual analogue scale (EQ‐VAS). The EQ‐5D‐5L has been widely tested and used in both general population and patient samples and has been translated into over 130 different language versions [www.euroqol.org].^18^ It has been stated in the guideline ‘each of the five dimensions comprising the EQ‐5D descriptive system is divided into five levels (scores) of perceived problems. Level/ score 1: indicating no problem; level/score 2: indicating slight problems; level/score 3: indicating moderate problems; level/score 4: indicating severe problems; level/score 5: indicating unable to/extreme problems’.^19^ For the current study we have defined level/score 1 as no problem and level/score 2–5 as having any impairment (mild to severe impairment) in each dimension regarding the HRQoL.

### 2.3 Medication adherence to anti‐diabetic drugs by Morisky Medication Adherence Scale

Morisky Scale (MMAS‐8) for medication adherence is an eight‐item standardized scale used widely to assess adherence to medication in chronic diseases.^15^, ^16^ The MMAS‐8 has been translated and adapted for use in people with diabetes in many countries.^20^, ^21^, ^22^ The MMAS‐8, contains eight questions with closed dichotomous (yes/no) answers, to understand the interviewee's adherence behaviour.^16^, ^23^ The degree of adherence was determined according to the score resulting from the sum of all the correct answers: high adherence (8 points), average adherence (6 to <8 points), and poor adherence (<6 points). For the current study, we have defined <6 scores as low adherence and 6–8 score as higher adherence group.

### 2.4 Covariates

The trained research technicians collected demographic data, medical history, use of medications, and lifestyle factors from the participants by the trained research technicians using a structured questionnaire. Body mass index (BMI) was defined as body weight (kg) divided by the square of the height (m). Using an automated sphygmomanometer (BP‐8800SF; Omron Health Care), the mean of two consecutive measurements on the right arm with participants in a seated position after a strict 5‐min rest period was used to determine blood pressure. Hypertension was defined as systolic blood pressure of ≥140 mm Hg, diastolic blood pressure of ≥90 mm Hg, or the use of antihypertensive medication.

All participants were diagnosed as having diabetes by the physicians in the facility and had a diabetes book that contains information about their current diabetes status (controlled or uncontrolled), most recent fasting blood sugar (FBS) value, and HBA1c value (for the last 3 months). Fasting blood sugar was measured from venous blood after 8 h of no calorie intake and 2 h of postprandial blood sugar was estimated by measuring venous blood 2 h after meal intake. The values for FBS, 2 h of postprandial blood sugar, or HbA1c were recorded in the patient's diabetes book as a routine check‐up at the facility, and the interviewers included the most recent values from their diabetes book. For the current study, uncontrolled diabetes mellitus was defined as HbA1c of >6.5%. The participants were also asked about the total duration of diabetes after diagnosis and were recorded.

The participants were also asked about their years of education, place of residence (rural, urban, and semi‐urban), occupation, monthly household expenditures, and any existing chronic diseases. We have defined multi‐morbidity as the co‐occurrence of at least one chronic disease with diabetes in participant,^9^ including the history of any sort of diagnosed heart disease, kidney disease, peripheral vascular disease, neurological insufficiencies, and chronic arthritis for our study participants.

### 2.5 Statistical analysis

The participants were sex‐stratified to compare the characteristics between the two groups. Participants' characteristics are shown using medians and interquartile ranges for continuous variables and percentages for categorical variables. The characteristics between the two groups were compared using the independent sample *t*‐test or Wilcoxon rank‐sum test for continuous variables and the χ^2^‐test for categorical variables. We also checked for a significant difference in five dimensions of quality of life (measured by EQ‐5D‐5L scoring) stratified by medication adherence status through the Cochrane–Mantel–Haenszel test for categorical variables. For the current study, we consider any impairment in all dimensions of (EQ‐5D‐5L) as our outcome and low adherence to anti‐diabetic medication as our exposure. Any impairment means if any participant has any problem ranging from mild to extreme problem or incapability to perform any work (score/level: 2 to 5) that falls into the following five dimensions; mobility, self‐care, usual activities, pain/discomfort, and anxiety/depression. Again we have included low adherence to anti‐diabetic drugs which is defined as a score less than <6 (the reference group is the higher adherence group, score 6–8).

Our analyses were performed with sequential adjustment: Model 1 was unadjusted, Model 2 was adjusted for age and sex, and Model 3 was simultaneously adjusted for all variables. Missing data were not included in the analyses. However, we also did an additional sex‐stratified multivariable logistic regression to assess any difference in finding for men and women groups. We used a masked data set and the outcome assessor was blinded. All analyses were performed by SAS version 9.4 (SAS Institute). A two‐tailed *p* value of <.05 was pre‐specified to indicate statistical significance.

## 3 RESULTS

### 3.1 The characteristics of the study participants

The mean age of the participants was 59.0 (standard deviation [SD], 7.0) years. The mean value of (EQ‐5D‐5L) scoring was 2.0 (SD, 1.0), and the mean value of EQ‐VAS scoring was 65.0 (SD, 15.1). Table 1 shows the proportions of five dimensions in five levels of EQ‐5D‐5L index for the participants and we found that the level of inabilities (problems) among the dimensions were different, for instance, 6.7%, 3.5%, 11.9%, 11.9% and 11.3% were reported to have a severe disability in terms of mobility, self‐care, usual daily activities, pain/discomfort, and anxiety respectively.

**TABLE 1:** Proportion of dimensions of EQ‐5D‐5L among people with diabetes in Dhaka, Bangladesh (*n* = 480).

Variable	No problems	Slight problems	Moderate problems	Severe problems	Extreme problems/unable to work
	*n* (%)	*n* (%)	*n* (%)	*n* (%)	*n* (%)
Mobility	271.0 (56.0)	88.0 (18.3)	79.0 (16.5)	32.0 (6.7)	10.0 (2.1)
Self‐care	327.0 (68.0)	88.0 (18.3)	36.0 (7.5)	17.0 (3.5)	12.0 (2.5)
Work, family, and leisure activities	288.0 (60.0)	99.0 (20.6)	54.0 (11.3)	27.0 (5.6)	12.0 (2.5)
Pain/discomfort	126.0 (26.3)	168.0 (35.0)	121.0 (25.2)	57.0 (11.9)	8.0 (1.7)
Anxiety/depression	109.0 (22.8)	174.0 (36.4)	133.0 (27.8)	54.0 (11.3)	8.0 (1.7)

*Note*: Data are expressed as frequency (*n*) and percentages (%). EQ‐5D‐5L dimensions were defined as the following codes; no problem = 1; slight problem = 2, moderate problem = 3; severe problem = 4; extreme problem/unable to work = 5.

Table 2 shows the characteristics of the participants stratified by sex. Men were elderly, had a higher level of education, higher household expenditure, lower blood glucose, higher duration of diabetes, lower waist and hip circumference, and a higher level of employment compared to women. Regarding the EQ‐5D‐5L index, women reported having more impaired mobility, impaired self‐care, and higher pain compared to the men and the differences were statistically significant. When stratified by levels of medication adherence (low, moderate, and high) to anti‐diabetic drugs, we also find that all the dimensions of EQ‐5D‐5L were statistically different among these groups(*p* < .01) (Table S1).

**TABLE 2:** Participants' characteristics stratified by sex among people with diabetes in Dhaka, Bangladesh (*n* = 480).

Characteristics	Men	Women	*p*‐Value*
*n* (%)	240.0 (50%)	240.0 (50%)	
Age, years	60.0 (55.0–65.0)	56.0 (52.0–62.0)	<.001
Education, years			
No formal education	20.0 (8.3)	46.0 (19.0)	<.001
1–5, years	41.0 (17.0)	59.0 (24.5)	
6–10, years	69.0 (28.7)	77.0 (32.0)	
11–12, years	37.0 (15.4)	35.0 (14.5)	
>12 years	73.0 (30.4)	23.0 (9.5)	
Place of residence			
Rural	46.0 (19.1)	46.0 (19.1)	.260
Urban	168.0 (70.0)	156.0 (65.0)	
Semi‐urban	26.0 (10.8)	38.0 (15.8)	
Self‐reported household expenditure, USD	372.1 (233.9–478.5)	265.8 (212.6–425.3)	.013
Fasting blood sugar, mmol/L	7.9 (6.4–10.1)	8.5 (6.5–11.4)	.012
2 h post prandial blood sugar, mmol/L	12.0 (9.2–14.7)	12.0 (9.1–15.8)	.292
Diabetes status			
Uncontrolled, (HbA1c >6.5)	161.0 (67.0)	152.0 (63.0)	.388
Diastolic BP, mmHg	80.0 (74.0–90.0)	80.0 (75.0–90.0)	.495
Systolic BP, mmHg	130.0 (120.0–140.0)	130.0 (120.0–140.0)	.291
Duration of diabetes, years	10.0 (6.0–16.0)	9.0 (5.0–14.5)	.011
BMI, kg/m^2^	25.2 (3.1)	26.4 (3.7)	.012
Waist circumference, cm	36.9 (3.4)	37.8 (4.0)	.037
Hip Circumference, cm	38.0 (4.4)	39.6 (5.2)	.014
Family history of diabetes, yes	241.0 (56.0)	26.0 (52.0)	.585
Medication adherence, (Morisky Scale Score)			
Non‐adherence	181.0 (75.4)	178.0 (74.0)	.561
Moderate adherence	44.0 (18.3)	51.0 (21.2)	
High adherence	15.0 (6.2)	11.0 (4.5)	
Hypertension, *n* (%)	100.0 (41.6)	111.0 (46.2)	.311
Occupation, *n* (%)			
Unemployed	99.0 (41.2)	18.0 (7.5)	<.001
Service holder	42.0 (17.5)	6.0 (2.5)	
Business	10.0 (4.1)	3.0 (1.5)	
Self‐employed	67.0 (27.9)	2.0 (0.8)	
Labourer	12.0 (5.0)	1.0 (0.4)	
Farmers	6.0 (2.5)	11.0 (4.5)	
House maker	4.0 (1.6)	199.0 (82.9)	
EQ‐5D‐5L scale			
Impaired Mobility, *n* (%)	86.0 (35.8)	123.0 (51.0)	<.001
Impaired Self‐care, *n* (%)	62.0 (25.8)	91.0 (37.9)	<.001
Impaired usual activities, *n* (%)	60.0 (12.5)	132.0 (27.5)	.563
Pain/Discomfort, *n* (%)	184.0 (76.6)	185.0 (77.0)	.022
Anxiety, *n* (%)	56.0 (23.3)	55.0 (22.9)	.913
EQ‐Vas Score (health today)	65.0 (55.0–75.0)	45.0 (40.0–55.0)	<.001
Multi‐morbidity, *n* (%)	176.0 (73.3)	159.0 (66.2)	.091

*Note*: Data are expressed as median (interquartile range) for skewed continuous variables and mean (SD) for normally distributed continuous variables or number (percentages) for categorical variables. The number of observations across the categories may not add up to the total given number because of missing data. Uncontrolled diabetes mellitus was defined as haemoglobin A1c of ≥6.5%. Hypertension was defined as systolic blood pressure of ≥140 mm Hg, diastolic blood pressure of ≥90 mm Hg, or the use of antihypertensive medication. Multi‐morbidity was defined as history of any sort of diagnosed heart disease, kidney disease, peripheral vascular disease, neurological insufficiencies, and chronic arthritis. EQ‐5D‐5L dimensions were defined as the following levels; no problem = 1; slight problem = 2, moderate problem = 3; severe problem = 4; unable = 6; Medication adherence was defined by Morisky Scale; high adherence (8 points), average adherence (6 to <8 points) and non‐adherence (low) (<6 points); SD = Standard deviation.

* Based on the independent sample *t*‐test for continuous variables with normal distribution /Wilcoxon rank‐sum test for continuous variables with skewed distribution and the χ^2^‐test for categorical variables.

### 3.2 Association between higher medication adherence to anti‐diabetic drugs and HRQoL (EQ‐5D‐5L index)

The results from multivariable logistic regression are shown in Table 3. Model 1, (unadjusted) showed that low adherence to anti‐diabetic medication (odds ratio [OR], 7.99; 95% confidence interval [CI], 1.07–12.9; *p* = .043) leads to a higher risk of anxiety. After adjusting by age and sex (Model 2), low adherence to anti‐diabetic medication (OR, 8.18; 95% CI, 1.10–15.9; *p* = .046) shows a stronger positive association with anxiety. In the final model after adjusting for age, sex, years of education, household expenditure, duration of diabetes, and the presence of comorbidities, we found low adherence to anti‐diabetic medication (OR, 0.26; 95% CI, 0.08–0.80; *p* = .036) interestingly shows lower risk of pain, but higher (OR, 7.18; 95% CI, 1.03–9.59; *p* = .043) risk of anxiety constant with other models.

**TABLE 3:** Multivariable logistic regression to assess the association between medication adherence and Quality of Life (EQ‐5D‐5L) impairment among people with diabetes in Dhaka, Bangladesh (*n* = 480).

	Quality of Life (EQ‐5D‐5L)				
	Impaired mobility (yes)	Impaired self‐care (yes)	Impaired usual activities (yes)	Pain/discomfort (yes)	Anxiety (yes)
	OR (95% CI)	OR (95% CI)	OR (95% CI)	OR (95% CI)	OR (95% CI)
Low adherence (vs. high adherence)					
Model 1	1.19 (0.50–2.47)	1.61 (0.72–3.59)	1.65 (0.37–2.25)	0.79 (0.33–1.86)	7.99* (1.07‐12.9)
Model 2	1.23 (0.54–2.76)	1.80 (0.79–4.09)	1.60 (0.27–2.25)	0.84 (0.35–2.02)	8.18* (1.10‐15.9)
Model 3	0.75 (0.30–1.90)	0.87 (0.35–2.18)	0.89 (0.40–1.97)	0.26* (0.08‐0.80)	7.18* (1.03‐9.59)

*Note*: Model 1 is unadjusted; Model 2 is adjusted for age and sex; Model 3 is adjusted for age, sex, years of education, household expenditure, hypertension, duration of diabetes, glycemic status, and multi‐morbidity.

* Data are odds ratio compared with the reference group for categorical variables. For the quality of life (EQ‐ 5D‐5L), we consider impairment ‘yes’ if slight problems to unable or/extreme problems (level 2–6) in case of mobility, self‐care, usual activities, pain/discomfort, anxiety, else ‘no’ impairment (score 1) which we consider as a reference; and for medication adherence, non‐adherence to anti‐diabetic drugs was defined as having score <6 in Morisky Scale and else adherence group (reference group) having a score between 6 and 8. Multi‐morbidity; history of any sort of diagnosed heart disease, kidney disease, peripheral vascular disease, neurological insufficiencies, and chronic arthritis.

Regarding the subgroup analysis in Table 4, low adherence to anti‐diabetic medication (OR, 0.20; 95% CI, 0.47–0.92; *p* = .044) showed a lower risk for pain in men, but no association among women was found. On the contrary, low adherence to anti‐diabetic medication reported a higher risk for anxiety in both men and women ([OR, 2.53; 95% CI, 1.07–13.0] and [OR, 2.50; 95% CI, 1.10–12.0], respectively).

**TABLE 4:** Multivariable logistic regression stratified by sex to assess the association between medication adherence and Quality of Life (EQ‐5D‐5L) impairment among people with diabetes in Dhaka, Bangladesh (*n* = 480).

	Quality of Life (EQ‐5D‐5L)					
		Impaired mobility (yes)	Impaired self‐care (yes)	Impaired usual activities (yes)	Pain/discomfort (yes)	Anxiety (yes)
		OR (95% CI)	OR (95% CI)	OR (95% CI)	OR (95% CI)	OR (95% CI)
Low adherence (vs. high adherence)						
Among men (*n* = 240)	Model	1.46 (0.40–5.29)	0.97 (0.25–3.68)	1.60 (0.27–2.25)	0.20* (0.47–0.92)	2.53* (1.07‐13.0)
Among women (*n* = 240)	Model	1.46 (0.40–5.29)	0.97 (0.25–3.68)	0.85 (0.32–2.45)	0.25 (0.39–1.01)	2.50* (1.10‐12.0)

*Note*: Model adjusted for age, sex, years of education, household expenditure, hypertension, duration of diabetes, glycemic status, and multi‐morbidity.

* Data are odds ratio compared with the reference group for categorical variables. For the quality of life (EQ‐ 5D‐5L), we consider impairment ‘yes’ if slight problems to unable or/extreme problems (level 2–6) in case of mobility, self‐care, usual activities, pain/discomfort, anxiety, else ‘no’ impairment (score 1) which we consider as a reference; and for medication adherence, non‐adherence to anti‐diabetic drugs was defined as having score <6 in Morisky Scale and else adherence group (reference group) having a score between 6 and 8. Multi‐morbidity; history of any sort of diagnosed heart disease, kidney disease, peripheral vascular disease, neurological insufficiencies, and chronic arthritis.

## 4 DISCUSSION

Bangladesh achieved remarkable economic growth of 7.24% in 2016–17 and new improvements have been added in the healthcare sectors, we believe it has its effects on the health‐related quality of life of the Bangladeshi population in recent years.^24^ In this particular study, we have discussed the association between the level of medication adherence and HRQoL among people with diabetes in Dhaka, Bangladesh. In this hospital‐based study, among HRQoL dimensions, higher medication adherence was significantly associated with lower anxiety symptoms and less severe pain. In clinical practice, the level of medication adherence to anti‐diabetic drugs might be monitored for a better HRQoL among people with diabetes. Our findings are also comparable with earlier studies with similar interest conducted among the diverse population with diabetes.^25^, ^26^, ^27^ Study reported adherent patients had significantly higher perceptions of Quality of life (QoL) and health when compared with non‐adherent patients.^26^

In this current study, we found all the participants had some sort of impairment in all the dimensions of the EQ‐5D‐5L index. In an earlier study conducted among older adults, most respondents had moderate scores of quality of life, and inadequate sleep, depression, religiosity, activities of daily living, and social support were key determinants for the quality of life.^7^ Another study demonstrated that health‐related quality of life among people with diabetes in Bangladesh is low, and various factors, such as socio‐demographic, psychological, and diabetes‐related complications were associated with it.^8^

Medication adherence is a major contributor to health status, and adherence rates differ considerably across many factors in chronic diseases. Identification of any substantial differences in adherence rates among people with diabetes is crucial for minimizing complications.^12^ Among people with diabetes, good adherence was associated with improved glycemic control, however, good adherence to diabetes medication remains an ongoing problem.^11^ An earlier systematic review reported that the proportion of adhering to diabetic medication ranges from 38.5% to 93.1%. Only 22.2% reported high adherence of ≥80% among the study population.^13^ Among South Asians including Bangladeshis, only 40%–45% were adherent to anti‐diabetic medications, including insulin and oral hypoglycemic agents.^28^ These findings are similar to our study results. In our study, we found a majority of the participants had a history of low adherence to anti‐diabetic medication (74%) and only a few (5%) had good (high) medication adherence to anti‐diabetic medication.

Regarding the association between medication adherence and quality of life, diabetes therapy, such as taking insulin or oral drugs can alter the quality of life either positively by lowering symptoms of higher blood sugar or negatively, by causing symptoms of lower blood sugar. Furthermore, getting medication for living with diabetes is often heavy, and can affect self‐care behaviour.^29^

EQ‐5D‐5L index has five dimensions, but we found only anxiety and pain to be associated with medication adherence, interestingly other dimensions like mobility, self‐care, and usual activity did not show any association with adherence level to anti‐diabetic drugs. Since we have included participants from a hospital set‐up, where people with diabetes usually come for routine check‐ups, a small proportion of them might have any activity‐related impairment, but anxiety is common. A nationwide cross‐sectional study among Bangladeshi adults found most of the HRQoL‐related impairment was due to anxiety (>70%).^30^ In this study, reported a positive association between anxiety and non‐adherence to anti‐diabetic medications. An earlier study reported a negative correlation between good adherence and diabetes‐related emotional distress.^31^ Understandably, good adherence to medication might have ensured higher assurance of less complication that reduces anxiety symptoms and leads to good medication adherence to chronic illness, therefore, lower HRQoL and anxiety were found to be positively associated with non‐adherence to appropriate medications in a population studied.^32^

Interestingly we found pain to be inversely associated with non‐adherence to anti‐diabetic medication in the current study. Pain is defined as ‘an unpleasant sensory and emotional experience associated with actual or potential tissue damage, or described in terms of such damage’.^33^ Therefore, pain is closely linked with linked psychological and social components. Usually, the presence of higher pain might cause a higher level of anxiety.^34^ Understandably, pain associated with anxiety has an immense impact on quality of life. Since medication can relieve pain sensations or anxiety symptoms, this approach could cause reverse effects.^35^ We believe the inverse association between low adherence and pain we reported, could be a cause of reverse casualty; less pain causes decreased pain‐related anxiety, therefore higher chances for low adherence to medication can happen, which we have reported in our study. However, due to a small‐scale cross‐sectional study design for our current study, we are unable to assess or confirm the fact of reverse casualty in this case. A future detailed study is recommended.

In sensitivity analysis, when we stratified by gender; we found similar findings in the case of men and women. In men, low adherence to anti‐diabetic medication showed an inverse association with pain and a positive association with anxiety whereas in women only anxiety shows a positive association. In the present study, men showed a higher proportion of adherence compared to women (Table 2). Since men adhere more to drugs than women, this could be a reason for the association between low adherence to anti‐diabetic drugs and pain, which we could not find among women.^36^ Nevertheless, further studies are recommended to understand this concern well, especially among people with diabetes.

Several limitations of the present study warrant consideration. First, we studied only participants obtained from a tertiary‐level specialized hospital in a single area in Dhaka, Bangladesh, and our participants were mostly walking patients with diabetes, who come to the facility for a routine check‐up and might have reported less HRQoL‐related impairments, which may limit the generalizability of the current results. Second, our study had a cross‐sectional study design, therefore, the true relationship between adherence to anti‐diabetic medication and quality of life might have been somewhat diluted. Third, local habits and practices for people with diabetes in this region were not included in this current study. Fourth, we do not have data for Type 1 and Type 2 diabetes separately and also unavailability of lipid profiles data, list of medications consumed by the study participants might have restricted detailed discussion about the quality of life depending on those factors. Despite some limitations, we believe, this study has generated new knowledge regarding the association between medication adherence and quality of life among people with diabetes in Bangladesh, and to our best knowledge, it would be the first study to assess such an association among people with diabetes in Bangladesh.

### 4.1 Clinical implication

In clinical practice, the level of medication adherence to anti‐diabetic drugs might be monitored for a better HRQoL among people with diabetes; however, it is necessary to understand whether improving medication adherence to anti‐diabetic drugs can improve HRQoL among patients with diabetes by conducting future additional research.

## 5 CONCLUSION

In a group of people with diabetes in Bangladesh, low medication adherence to anti‐diabetic drugs shows an association with the presence of anxiety and pain among the five dimensions of the quality of life index (EQ‐5D‐5L). This study emphasizes that medication adherence to be addressed to ensure the overall well‐being of people with diabetes. This study reported on the importance of medication adherence to contribute to the quality of life; however, further studies are recommended.

## AUTHOR CONTRIBUTIONS

**Sabrina Ahmed:** Conceptualization (equal); data curation (equal); formal analysis (equal); funding acquisition (equal); investigation (equal); methodology (equal); project administration (equal); writing – original draft (equal); writing – review and editing (equal). **KM Saif‐Ur Rahman:** Conceptualization (equal); investigation (equal); methodology (equal); writing – review and editing (equal). **Raja Ram Dhungana:** Conceptualization (equal); investigation (equal); methodology (equal); writing – review and editing (equal). **Gantsetseg Ganbaatar:** Conceptualization (equal); investigation (equal); methodology (equal); writing – review and editing (equal). **Fatema Ashraf:** Conceptualization (equal); funding acquisition (equal); methodology (equal); project administration (equal); writing – review and editing (equal). **Yuichiro Yano:** Conceptualization (equal); methodology (equal); supervision (equal); writing – review and editing (equal). **Katsuyuki Miura:** Conceptualization (equal); methodology (equal); supervision (equal); writing – review and editing (equal). **M. S. A. Mansur Ahmed:** Conceptualization (equal); funding acquisition (equal); investigation (equal); methodology (equal); project administration (equal); supervision (equal); writing – review and editing (equal).

## CONFLICT OF INTEREST STATEMENT

The authors have no conflicts of interest to declare.

## Supporting information


Table S1.

## Data Availability

The data of deidentified patients are not publicly available and will not be shared due to privacy or ethical restrictions.
