# Supplementary material for: Medication adherence and health‐related quality of life among people with diabetes in Bangladesh: A cross‐sectional study
Source: Endocrinol Diabetes Metab. 2023 Jul 25;6(5):e444. doi: 10.1002/edm2.444 (PMC10495558; doi:10.1002/edm2.444)
Supplement: Supplementary file 1 — Table S1. [file EDM2-6-e444-s001.docx]

**Supplementary Table S1**

Status of Health related Quality of Life (EQ-5D-5L) impairment stratified by levels of medication adherence among people with diabetes in Dhaka, Bangladesh (n=480)

| Variable | | Low  adherence | | Medium Adherence | | High Adherence | | Total | | * P value |
| --- | --- | --- | --- | --- | --- | --- | --- | --- | --- | --- |
|  |  | n | % | n | % | n | % | n | % |  |
| Health status (EQ- 5D- 5L) – Mobility* | No problems in walking | 213 | 59.3 | 44 | 46.3 | 14 | 53.8 | 271 | 56.5 | 0.174 |
|  | Slight problems with walking | 67 | 18.7 | 18 | 18.9 | 3 | 11.5 | 88 | 18.3 |  |
|  | Moderate problems with walking | 53 | 14.8 | 21 | 22.1 | 5 | 19.2 | 79 | 16.5 |  |
|  | Severe problems with walking | 20 | 5.6 | 9 | 9.5 | 3 | 11.5 | 32 | 6.7 |  |
|  | Unable to walk | 6 | 1.7 | 3 | 3.2 | 1 | 3.8 | 10 | 2.1 |  |
| Health status (EQ- 5D- 5L) – Selfcare* | No problems washing or dressing myself | 262 | 73.0 | 50 | 52.6 | 15 | 57.7 | 327 | 68.1 | <0.01 |
|  | Slight problems washing or dressing myself | 56 | 15.6 | 27 | 28.4 | 5 | 19.2 | 88 | 18.3 |  |
|  | Moderate problems washing or dressing myself | 24 | 6.7 | 9 | 9.5 | 3 | 11.5 | 36 | 7.5 |  |
|  | Severe problems washing or dressing myself | 9 | 2.5 | 5 | 5.3 | 3 | 11.5 | 17 | 3.5 |  |
|  | Unable to wash or dress myself | 8 | 2.2 | 4 | 4.2 | 0 | 0.0 | 12 | 2.5 |  |
| Health status (EQ- 5D- 5L) - Work, study, housework, family, and leisure activities* | Problems doing my usual activities | 234 | 65.2 | 41 | 43.2 | 13 | 50.0 | 288 | 60.0 | <0.01 |
|  | Slight problems doing my usual activities | 71 | 19.8 | 25 | 26.3 | 3 | 11.5 | 99 | 20.6 |  |
|  | Moderate problems doing my usual activities | 32 | 8.9 | 18 | 18.9 | 4 | 15.4 | 54 | 11.3 |  |
|  | Severe problems doing my usual activities | 16 | 4.5 | 5 | 5.3 | 6 | 23.1 | 27 | 5.6 |  |
|  | Unable to do my usual activities | 6 | 1.7 | 6 | 6.3 | 0 | 0.0 | 12 | 2.5 |  |
| Health status (EQ- 5D- 5L) - Pain/discomfort* | No pain or discomfort | 108 | 30.1 | 10 | 10.5 | 8 | 30.8 | 126 | 26.3 | <0.01 |
|  | Slight pain or discomfort | 124 | 34.5 | 42 | 44.2 | 2 | 7.7 | 168 | 35.0 |  |
|  | Moderate pain or discomfort | 81 | 22.6 | 30 | 31.6 | 10 | 38.5 | 121 | 25.2 |  |
|  | Severe pain or discomfort | 39 | 10.9 | 12 | 12.6 | 6 | 23.1 | 57 | 11.9 |  |
|  | Extreme pain or discomfort | 7 | 1.9 | 1 | 1.1 | 0 | 0.0 | 8 | 1.7 |  |
| Health status (EQ- 5D- 5L) - Anxiety/depression* | Anxious or depressed | 99 | 27.7 | 9 | 9.6 | 1 | 3.8 | 109 | 22.8 | <0.01 |
|  | Slightly anxious or depressed | 129 | 36.0 | 40 | 42.6 | 5 | 19.2 | 174 | 36.4 |  |
|  | Moderately anxious or depressed | 96 | 26.8 | 30 | 31.9 | 7 | 26.9 | 133 | 27.8 |  |
|  | Severely anxious or depressed | 29 | 8.1 | 14 | 14.9 | 11 | 42.3 | 54 | 11.3 |  |
|  | Extremely anxious or depressed | 5 | 1.4 | 1 | 1.1 | 2 | 7.7 | 8 | 1.7 |  |

Data are expressed as numbers (percentages) for categorical variables. The number of observations across the categories may not add up to the total given number because of missing data.

*P value Based on Cochran-Mantel-Haenszel test (<0.05 as level of significance)
